# A rapid, specific, extraction-less, and cost-effective RT-LAMP test for the detection of SARS-CoV-2 in clinical specimens

**DOI:** 10.1371/journal.pone.0266703

**Published:** 2022-04-11

**Authors:** Francesco Elia Marino, Eric Proffitt, Eugene Joseph, Arun Manoharan

**Affiliations:** Department of Research and Development, Prime Discoveries INC, Philadelphia, Pennsylvania, United States of America; University of Helsinki: Helsingin Yliopisto, FINLAND

## Abstract

In 2019 a newly identified coronavirus, designated as severe acute respiratory syndrome coronavirus 2 (SARS-CoV-2), has spread rapidly from the epicenter in Wuhan (China) to more than 150 countries around the world, causing the Coronavirus disease 2019 (COVID-19) pandemic. In this study, we describe an extraction-less method based on reverse transcriptase loop-mediated isothermal amplification (RT-LAMP) intended for the rapid qualitative detection of nucleic acid from SARS-CoV-2 in upper respiratory specimens, including oropharyngeal and nasopharyngeal swabs, anterior nasal and mid-turbinate nasal swabs, nasopharyngeal washes/aspirates or nasal aspirates as well as bronchoalveolar lavage (BAL) from individuals suspected of COVID-19 by their healthcare provider. The assay’s performance was evaluated and compared to an RT quantitative PCR-based assay (FDA-approved). With high sensitivity, specificity, and bypassing the need for RNA extraction, the RT-LAMP Rapid Detection assay is a valuable and fast test for an accurate and rapid RNA detection of the SARS-CoV-2 virus and potentially other pathogens. Additionally, the versatility of this test allows its application in virtually every laboratory setting and remote location where access to expensive laboratory equipment is a limiting factor for testing during pandemic crises.

## Introduction

In December 2019, an outbreak in Wuhan of a severe respiratory illness was caused by a previously unrecognized coronavirus, which has since been named severe acute respiratory syndrome coronavirus 2 (SARS-CoV-2) [1–6]. After the virus spread in more than 150 countries worldwide, the COVID-19 was declared a worldwide pandemic. The SARS-CoV-2 related pandemic has posed challenges for the global market, economy, and scientific research and underlined significant inequality and inaccessibility to testing for many countries worldwide [7,8]. Point-of-care serial screening can provide rapid results, and it is critical to identify asymptomatic individuals carrying the virus. Classic methods of screening, and virus RNA detection like RT-PCR, are labor expensive, require additional reagents for RNA extraction, and highly trained technicians in molecular biology techniques are needed. RT-LAMP (loop-mediated isothermal amplification) methods based on a colorimetric read-out have been evaluated as a suitable alternative to the regular PCR methods [9]. However, samples require refrigeration and must be analyzed within a short time frame. Additionally, RT-LAMP combined with a colorimetric read-out poses challenges for data interpretation due to high ambiguity and pH fluctuations can easily alter the readout [10].

Additionally, the current pandemic has introduced an unprecedented challenging situation in obtaining plastic consumables and RNA extraction kits. Manufacturers worldwide still have issues satisfying the demand for highly requested reagents for SARS-CoV-2 serial screening and biomedical research in general. The crisis has also accentuated significant disparities, and laboratories in remote locations and with limited budgets can hardly afford expensive quantitative PCR equipment and reagents. Rapid tests for SARS-CoV-2 were implemented as fast assays to control transmission and provide results in less than one hour. However, increasing evidence suggests significant risks associated with the false positivity and negativity rate of these tests, especially if the screening strategy is based on lateral flow antigen tests (rapid tests) [11,12]. Thus, alternative testing methods based on fast and reliable approaches without compromising the rigor of the testing pipeline are critically needed.

The current study shows an extraction-less method that can go from patient sample collection to testing and data interpretation in less than one hour (Prime CovidDetect^™^ Rapid Detection kit). This method is based on isothermal amplification and can be performed virtually on every equipment able to maintain a temperature of 65 degrees Celsius for 50 minutes and can be paired with any plate reader and does not necessarily need quantitative PCR equipment for data interpretation and visualization. The assay utilizes the well-established LAMP (loop-mediated isothermal amplification) method [6,13–15] combined with the innovative iSWAB^™^ Extraction-less buffer (Mawi DNA Technologies) that was designed to eliminate the RNA extraction step in the COVID-19 Molecular testing workflow, allowing researchers to perform direct RT-PCR or RT-LAMP on individual and pooled samples. The iSWAB- Extraction-less buffer is a non-toxic stabilizing technology that enables the inactivation of bacteria, fungi, spores, and viruses, allows ambient collection and transport of various bio-samples, and preserve the nucleic acid material at the time of collection. The buffer stabilizes nasal swabs, and these samples can be used directly in the RT-LAMP reactions without any prior major (RNA extraction) or minor (heating or/and Proteinase K treatment) sample processing and successfully detect SARS-CoV-2 without any observed PCR inhibition. After assessing the Limit of Detection (LoD) and comparing the detection rate, sensitivity, specificity, of the Prime CovidDetect^™^ Rapid Detection kit to a standard comparator assay (FDA approved) for the detection of SARS-CoV-2, we showed that the assay is a robust alternative to PCR based assays and can be virtually adopted in any laboratory settings for the rapid identification of the SARS-CoV-2 RNA.

## Material and methods

### LAMP reagents and reaction set-up

The Prime CovidDetect^™^ Rapid Detection kit used eighteen primers to identify SARS-CoV-2 (ORF1 a/b, E, and N genes) and six primers to identify the 18S Ribosomal RNA gene (18S RNA) used as control. The primers’ sequences are illustrated in Table 1. Primers were mixed to obtain the following final concentrations per reaction (FIP 0.8 μM, BIP 0.8 μM, F3 0.1 μM, B3 0.1 μM, LB 0.2 μM, LF 0.2 μM). LAMP enzyme and Dye were purchased from New England Biolabs (cat# E1700L). The reaction set-up was prepared as follows: 4 μl of Enzyme Mix, 0.5 μl of SARS-CoV-2 Primers Mix (from 20X Stock) or 0.5 μl of 18S RNA Primers Mix (from 20X Stock), 0.25 μl of LAMP Dye, 3 μl of Input RNA (from the iSWAB^™^ Extraction-less buffer), 2.25 of Nuclease-Free Water (final reaction volume was 10 μl).

**Table 1:** LAMP primers.

Primer Name	Sequence (5’-3’)
**N Gene**	
N2-F3	ACCAGGAACTAATCAGACAAG
N2-B3	GACTTGATCTTTGAAATTTGGATCT
N2-FIP	TTCCGAAGAACGCTGAAGCGGAACTGATTACAAACATTGGCC
N2-BIP	CGCATTGGCATGGAAGTCACAATTTGATGGCACCTGTGTA
N2-LF	GGGGGCAAATTGTGCAATTTG
N2-LB	CTTCGGGAACGTGGTTGACC
**E Gene**	
E1-F3	TGAGTACGAACTTATGTACTCAT
E1-B3	TTCAGATTTTTAACACGAGAGT
E1-FIP	ACCACGAAAGCAAGAAAAAGAAGTTCGTTTCGGAAGAGACAG
E1-BIP	TTGCTAGTTACACTAGCCATCCTTAGGTTTTACAAGACTCACGT
E1-LF	CGCTATTAACTATTAACG
E1-LB	GCGCTTCGATTGTGTGCGT
**ORF1 gene**	
ORF1-F3	CGGTGGACAAATTGTCAC
ORF1-B3	CTTCTCTGGATTTAACACACTT
ORF1-FIP	TCAGCACACAAAGCCAAAAATTTATTTTTCTGTGCAAAGGAAATTAAGGAG
ORF1-BIP	TATTGGTGGAGCTAAACTTAAAGCCTTTTCTGTACAATCCCTTTGAGTG
ORF1-LF	TTACAAGCTTAAAGAATGTCTGAACACT
ORF1-LB	TTGAATTTAGGTGAAACATTTGTCACG
**18S RNA**	
18S RNA-F3	GTTCAAAGCAGGCCCGAG
18S RNA-B3	CCTCCGACTTTCGTTCTTGA
18S RNA-FIP	TGGCCTCAGTTCCGAAAACCAACCTGGATACCGCAGCTAGG
18S RNA-BIP	GGCATTCGTATTGCGCCGCTGGCAAATGCTTTCGCTCTG
18S RNA-LF	AGAACCGCGGTCCTATTCCATTATT
18S RNA-LB	ATTCCTTGGACCGGCGCAAG

For each sample, four reactions were prepared: two replicates were prepared to detect SARS-CoV-2 and two for the detection of 18S RNA. Samples were loaded into a 384-well plate (cat#4309849 Thermo Fisher Scientific), sealed with optical adhesive film (cat# 4311971 Fisher Scientific), and centrifuged at 1000 rpm for 1 minute. The QuantStudio^™^ 5 was used to set up the reaction as follows: 100 cycles (each cycle of 30 seconds incubated at 65 degrees Celsius) were selected as PCR steps (on the FAM channel), and data collection was set to ON (for data collection). Cut-off values were applied as illustrated in Table 2. See the S1 File for a step-by-step instrument set-up and alternative instruments that could be used with the assay.

**Table 2:** Cut-off values.

Control Type	RT LAMP	
	ORF1, E, N (FAM channel)	18S RNA (FAM channel)
Negative	Non-detected or detection ≥ 80 cycles	Non-detected or detection ≥ 80 cycles
Positive	Detection ≤ 80 cycles	Detection ≤ 80 cycles

### Analytical sensitivity

Quantified heat-inactivated SARS-CoV-2 virus (cat#VR-1986 ATCC Lot# 70042082–3.9 x 10^5^ genome copies/ml) was spiked into a real clinical matrix (nasopharyngeal swabs from 10 negative samples collected in iSWAB^™^ Extraction-less buffer) and used for serial dilutions. The LoD concentration was determined by testing 24 individual replicates for different dilutions (as recommended by the FDA). LoD was defined as the lowest concentration at which more than 95% of replicates were positive. Replicates were called negative if no amplification was detected before cycle 80 (threshold value established based on nonspecific amplification observed for detection at cycles ≥ 80) of the RT-LAMP according to the assay selecting criteria to call a sample positive or negative. Homology analysis was conducted for the ORF1, E, and N, primer sets against all SARS-CoV-2 sequences deposited at GISAID [16–18] on March 16, 2022. A total of 9,308,692 sequences were considered, of which 3,535,497 were discarded for being incomplete (≤ 29kb) or having poor coverage (≥ 1% undefined bases). The remaining 5,773,195 sequences comprise a superset of those sequences considered by GISAID to be both complete and high-coverage (GISAID evaluates genomes >29,000bp as complete and further assigns labels of high coverage <1% Ns—undefined bases- and low coverage >5% Ns. The exact locations of the primer regions in each sequence were identified from the multiple sequence alignment file provided by GISAID. Subsequently, for each of the three primer sets, the number of mismatches per sequence was calculated using the Levenshtein distance metric [19] Table 3.

**Table 3:** In silico inclusivity analysis.

	N-gene	E-gene	ORF1 region
**Total Primer Length (nt)**	169	168	187
**Total # of Strains Evaluated**	5773195	5773195	5773195
**100% Match**	5341972	4612424	5473635
**1 Mismatch**	409281	1147905	276374
**2 Mismatches**	17108	7490	17235
**3 Mismatches**	919	128	637
**>3 Mismatches**	3915	5248	5314

### Analytical specificity

In silico cross-reactivity analysis was performed by aligning the SARS-CoV-2 primer sequences against sequences of common viruses as well as those coronaviruses most closely related to SARS-CoV-2. See Table 4 for the organisms assessed in silico for potential cross-reactivity. The analytical specificity was also assessed by wet testing. Briefly, samples were prepared by spiking intact viral particles or cultured RNA or bacterial cells into real clinical matrix as described before using panels/organisms from Zeptometrix, BEI Resources, and ATCC Table 5. Because no quantification information was available for the individual wet tested organisms, 50 μL of each stock was spiked into a negative clinical matrix and tested in replicates of three.

**Table 4:** *In Silico* cross-reactivity/exclusivity.

GenBank	Designation	N-gene	E-gene	Orf1 region
MN908947.3	Severe acute respiratory syndrome coronavirus 2 isolate Wuhan-Hu-1, complete genome	100.00%	100.00%	100.00%
NC_002645.1	Human coronavirus 229E, complete genome	56.80%	54.80%	47.60%
NC_006213.1	Human coronavirus OC43 strain ATCC VR-759, complete genome	49.70%	48.80%	44.40%
NC_006577.2	Human coronavirus HKU1, complete genome	46.20%	50.00%	48.70%
NC_005831.2	Human Coronavirus NL63, complete genome	57.40%	55.40%	48.10%
NC_004718.3	SARS coronavirus Tor2, complete genome	82.20%	96.40%	44.40%
NC_019843.3	Middle East respiratory syndrome-related coronavirus isolate HCoV-EMC/2012, complete genome	48.50%	54.20%	47.60%
X67709.1	Adenovirus type 1 hexon gene	13.60%	13.70%	31.00%
NC_039199.1	Human metapneumovirus isolate 00–1, complete genome	43.80%	54.80%	47.10%
AF457102.1	HPIV-1 strain Washington/1964, complete genome	53.80%	50.60%	48.10%
AF533012.1	Human parainfluenza virus 2 strain GREER, complete genome	45.00%	50.00%	49.20%
KF530234.1	Human parainfluenza virus 3 strain HPIV3/MEX/1526/2005, complete genome	48.50%	50.60%	46.50%
NC_021928.1	Human parainfluenza virus 4a viral cRNA, complete genome, strain: M-25	46.70%	54.80%	45.50%
FJ966079.1	Influenza A virus (A/California/04/2009(H1N1)) segment 1 polymerase PB2 (PB2) gene, complete cds	44.40%	34.50%	34.20%
KT002533.1	Influenza A virus (A/canine/Illinois/12191/2015(H3N2)) segment 1 polymerase PB2 (PB2) gene, complete cds	37.30%	32.70%	23.50%
MN230203.1	Influenza B virus (B/California/24/2019) segment 1 polymerase PB1 (PB1) gene, complete cds	29.00%	23.80%	29.40%
MK715533.1	Influenza B virus (B/California/40/2018) segment 1 polymerase PB1 (PB1) gene, complete cds	35.50%	41.70%	37.40%
KP745766.1	Enterovirus D68 isolate NY328, complete genome	45.00%	41.70%	42.80%
U39661.1	Respiratory syncytial virus, complete genome	49.70%	50.00%	50.30%
NC_001490.1	Rhinovirus B14, complete sequence	45.60%	44.60%	44.40%

**Table 5:** Cross-reactivity/exclusivity wet testing of the Prime CovidDetect^™^ rapid detection kit.

Organism	Strain	Provider	Catalog number	ORF1/N/E-gene Detected Replicates
Adenovirus 11	Slobitski	ATCC	VR-12	0/3
Adenovirus 5	Adenoid 75	ATCC	VR-5	0/3
*Bordetella pertussis*	18323 [NCTC 10739]	ATCC	9797	0/3
*Candida albicans*	NIH 3172	ATCC	14053	0/3
*Chlamydophila pneumoniae*	TWAR strain 2023	ATCC	VR-1356	0/3
Enterovirus 70	J670/71	ATCC	VR-836	0/3
*Haemophilus influenzae*	NCTC 8143	ATCC	33391	0/3
Human parainfluenza virus 4b	CH 19503	ATCC	VR-1377	0/3
Human respiratory syncytial virus	A2	ATCC	VR-1540P	0/3
Human rhinovirus 61	6669-CV39 [V-152-002-021]	ATCC	VR-1171	0/3
*Mycobacterium tuberculosis*	H37Ra	ATCC	25177	0/3
*Mycoplasma pneumoniae*	Somerson et al. FH strain of Eaton Agent [NCTC 10119]	ATCC	15531	0/3
*Pseudomonas aeruginosa*	(Schroeter) Migula (ATCC^®^ 10145^™^)—[CCEB 481, MDB strain BU 277, NCIB 8295, NCPPB 1965, NCTC 10332, NRRL B-771, R. Hugh 815]	ATCC	10145	0/3
*Staphylococcus epidermidis*	AmMS 205	ATCC	49134	0/3
*Streptococcus pneumoniae*	Mu50 [NRS1]	ATCC	700699	0/3
*Streptococcus pyogenes*	Rosenbach (ATCC^®^ 49399^™^–QC A62)	ATCC	49399	0/3
*Streptococcus salivarius*	B2	ATCC	9759	0/3
Human coronavirus		BEI	NL63	0/3
Human coronavirus		BEI	229E	0/3
Human coronavirus, Middle East Respiratory Syndrome Coronavirus (MERS-CoV),	EMC/2012	BEI	NR-50549	0/3
SARS Coronavirus		BEI	NR-3882	0/3
SARS-Related Coronavirus 2		BEI	NR-52286	0/3
A. baumannii	307–0294	ZeptoMetrix	NATPPQ-BIO	0/3
Adenovirus Type 3		ZeptoMetrix	NATRVP-1	0/3
Adenovirus Type 3		ZeptoMetrix	NATPPA-BIO	0/3
Adenovirus Type 31		ZeptoMetrix	NATPPA-BIO	0/3
C. pneumoniae	CWL-029	ZeptoMetrix	NATPPA-BIO	0/3
Coronavirus	229E	ZeptoMetrix	NATRVP-1	0/3
Coronavirus	NL63	ZeptoMetrix	NATPPA-BIO	0/3
Coronavirus	OC43	ZeptoMetrix	NATRVP-1	0/3
Coronavirus	SARS	ZeptoMetrix	NATRVP-1	0/3
E. cloacae	Z101	ZeptoMetrix	NATPPQ-BIO	0/3
E. coli	Z297	ZeptoMetrix	NATPPQ-BIO	0/3
Enterovirus		ZeptoMetrix	NATRVP-1	0/3
H. influenzae	MinnA	ZeptoMetrix	NATPPQ-BIO	0/3
Human Metapneumovirus		ZeptoMetrix	NATRVP-1	0/3
Influenza A	H1	ZeptoMetrix	NATRVP-1	0/3
Influenza A	H1N1 (2009)	ZeptoMetrix	NATRVP-1	0/3
Influenza A	H3	ZeptoMetrix	NATRVP-1	0/3
Influenza A	H3 A/Brisbane/10/07	ZeptoMetrix	NATPPA-BIO	0/3
Influenza B		ZeptoMetrix	NATRVP-1	0/3
Influenza B	B/Florida/02/06	ZeptoMetrix	NATPPA-BIO	0/3
K. aerogenes	Z052	ZeptoMetrix	NATPPQ-BIO	0/3
K. oxytoca	Z115	ZeptoMetrix	NATPPQ-BIO	0/3
K. pneumoniae	KPC2	ZeptoMetrix	NATPPQ-BIO	0/3
K. pneumoniae	Z138; OXA-48	ZeptoMetrix	NATPPQ-BIO	0/3
K. pneumoniae	Z460; NDM-1	ZeptoMetrix	NATPPQ-BIO	0/3
L. pneumophila	Philadelphia	ZeptoMetrix	NATPPA-BIO	0/3
M. catarrhalis	Ne 11	ZeptoMetrix	NATPPQ-BIO	0/3
M. pneumoniae	M129	ZeptoMetrix	NATPPA-BIO	0/3
Metapneumovirus	8 Peru6-2003	ZeptoMetrix	NATPPA-BIO	0/3
P. aeruginosa	Z139, VIM-1	ZeptoMetrix	NATPPQ-BIO	0/3
P. mirabilis	Z050	ZeptoMetrix	NATPPQ-BIO	0/3
Parainfluenza virus Type 1		ZeptoMetrix	NATPPA-BIO	0/3
Parainfluenza virus Type 1		ZeptoMetrix	NATRVP-1	0/3
Parainfluenza virus Type 2		ZeptoMetrix	NATRVP-1	0/3
Parainfluenza virus Type 3		ZeptoMetrix	NATRVP-1	0/3
Respiratory Syncytial Virus A		ZeptoMetrix	NATRVP-1	0/3
Respiratory Syncytial Virus B		ZeptoMetrix	NATRVP-1	0/3
Rhinovirus 1A		ZeptoMetrix	NATRVP-1	0/3
Rhinovirus 1A		ZeptoMetrix	NATPPA-BIO	0/3
RSV A2		ZeptoMetrix	NATPPA-BIO	0/3
S. agalactiae	Z019	ZeptoMetrix	NATPPQ-BIO	0/3
S. aureus	MRSA;COL	ZeptoMetrix	NATPPQ-BIO	0/3
S. marcescens	Z053	ZeptoMetrix	NATPPQ-BIO	0/3
S. pneumoniae	Z022	ZeptoMetrix	NATPPQ-BIO	0/3
S. pyogenes	Z018	ZeptoMetrix	NATPPQ-BIO	0/3

### Clinical samples

Positive (n = 30) and negative (n = 34) nasopharyngeal swabs were purchased from LEE BioSolutions and placed in the MAWI iSWAB^™^ Extraction-less buffer. The manufacturer confirmed samples’ negative or positive status using the TaqPath COVID-19 combo kit (cat# A47814 Thermo Fisher Scientific). The samples’ status (negative or positive) was re-confirmed by using the FDA-approved Quick-SARS-CoV-2 rRT-PCR kit (cat# R3011 Zymo Research) following the manufacturer’s instructions. According to the manufacturer’s provided information, the symptomatic status of the patients was unknown at the time of collection. Thus, an additional set of samples with known patients’ symptomatic status, positive symptomatic (n = 32), positive asymptomatic (n = 36), and negative (n = 49) were obtained from a diagnostic lab (Hook Diagnostics) and re-confirmed using the Quick-SARS-CoV-2 rRT-PCR kit (cat# R3011 Zymo Research). These samples were collected from 7 testing sites across the United States.

### RNA extraction

For samples to be analyzed with the comparator assay (Quick-SARS-CoV-2 rRT-PCR kit), 140 μL of input material (nasopharyngeal swab in iSWAB^™^ extraction-less MAWI buffer) was used for RNA extraction performed with the QIAamp Viral RNA kit (cat# 52906 Qiagen) according to the manufacturer’s instructions except for the final elution step (performed in 20 μL of AVE buffer instead of 60 μL).

## Results

### Sensitivity and specificity of the assay

**Fig 1:**
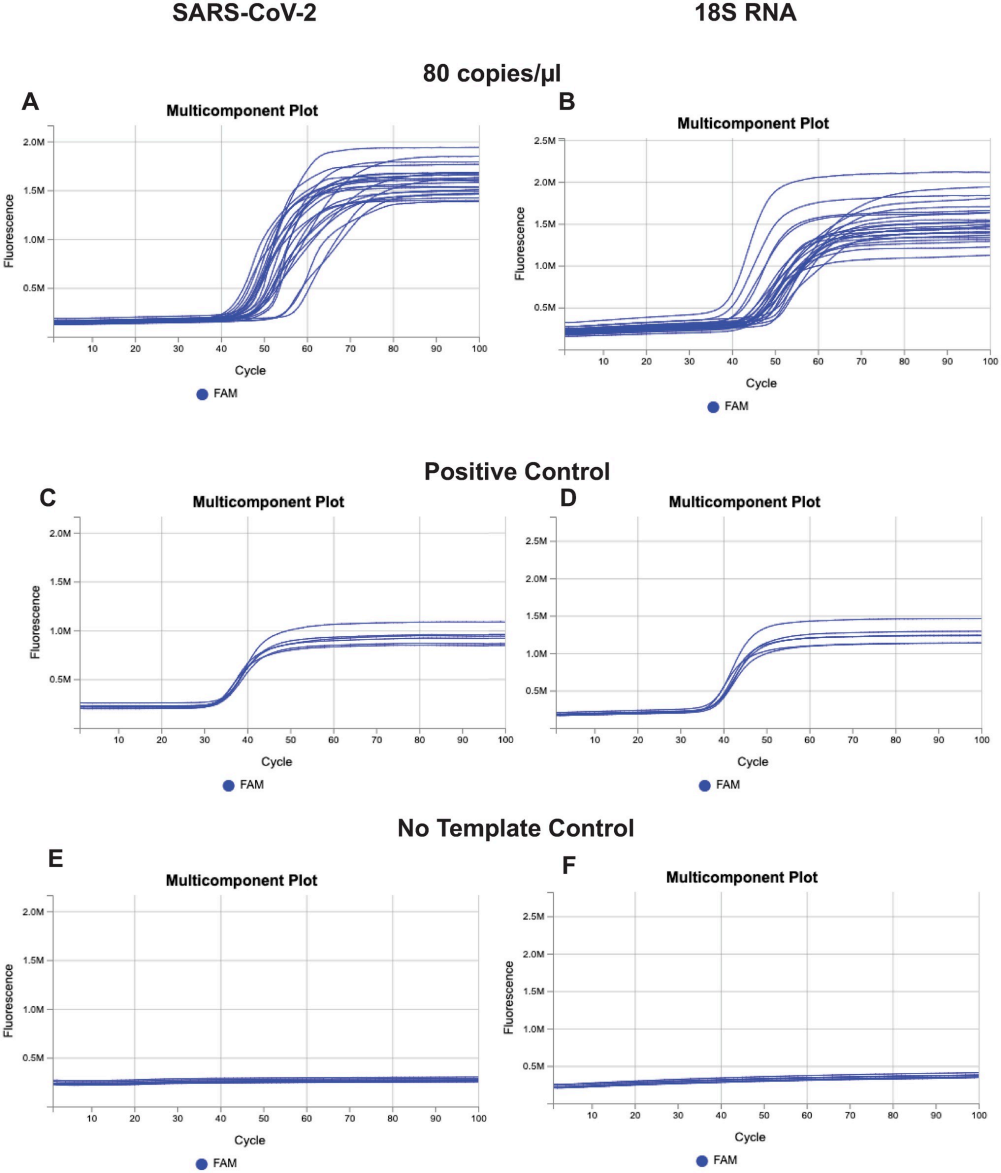
Amplification plots for SARS-CoV-2 and 18S RNA.

To assess the sensitivity of the assay we firstly investigated the limit of detection (LoD) to define the lowest limit at which the assay can detect the presence of intact virus with consistency and reproducibility. The LoD determination of the Prime CovidDetect^™^ Rapid Detection kit was 80 copies/μL, Table 6. The amplification plots of the 24 replicate wells for SARS-CoV-2 are shown in Fig 1; specifically, Orf1, E1, N2 genes (Fig 1A), and the 18S RNA control gene (Fig 1B). The positive control (Fig 1C and 1D) was SARS-CoV-2 (heat-inactivated and spiked as previously described) at the dilution at 10,000 copies/μL (six replicates) and the No Template Control (Fig 1E and 1F) was Nuclease-Free water (6 replicates).

**Table 6:** Limit of detection (LoD) of the Prime CovidDetect^™^ rapid detection kit.

Concentration	ORF1/E/N
Copies/μl	(Replicates detected)
1000	24/24 (100%)
100	24/24 (100%)
**80**	**24/24 (100%)**
70	22/24 (91.7%)
50	19/24 (79.1%)
10	11/24 (41.7%)

We then proceeded with a bioinformatic analysis to identify if the primers used within the assay were specific for SARS-CoV-2. Each primer set matched at 100% similarity against the SARS-CoV-2 Ref Seq reference genome (Wuhan-Hu-1; NC_045512.1) Table 3. In addition, an in-silico inclusivity analysis determined that the N primer set differed by one or fewer mutations for approximately 99.6% of GISAID sequences, the E primer set for 99.8%, and the ORF1 primer set for 99.8%. In total, it was determined that only 470 GISAID sequences differed by more than one nucleotide for two out of three SARS-CoV-2 primer sets, and only 41 sequences differed by more than one nucleotide for all three. Indeed, the potential for poor primer hybridization to co-occur across all three primer sets is exceedingly rare, at approximately 1 in 140,810. Our analysis revealed that the primers used to detect the genes Orf1, E, and N of the SARS-CoV-2 virus are highly specific (SARS-CoV-2 Gene Bank Reference MN908947.3) and show minimal cross-reactivity with other coronaviruses, adenoviruses, or influenza viruses Table 4. Additionally, as shown in Table 5, when the assay was used in wet testing for pathogens similar or related to SARS, no replicates were detected. Thus, both in silico and wet testing analysis showed a high specificity of the Prime CovidDetect^™^ Rapid Detection assay.

### Clinical evaluation

To determine the detection rate of both positive and negative confirmed nasopharyngeal swabs we assessed the performance evaluation of the Prime CovidDetect^™^ Rapid Detection kit on clinical samples. Samples from two sources, and from symptomatic or asymptomatic patients were used. Additionally, samples were obtained from several testing sites across the United States to consider patients’ variability and potential differences in collection methods. When compared to a comparator test, approved by the FDA, the Prime CovidDetect^™^ Rapid Detection test showed a 100% detection rate Table 7. Positive Samples included a total of 24 out of 98 samples with a Ct value > 30 (clinically challenging samples) as quantified by the comparator assay (S2 File).

**Table 7:** Evaluation of clinical samples and comparison to the Zymo FDA-Approved comparator assay.

	Prime CovidDetect^™^		FDA Approved Comparator Assay		% Agreement
	Positive	Negative	Positive	Negative	
**Positive Unknown Status**	**30**		**30**		**100%**
**Positive Symptomatic**	**32**		**32**		**100%**
**Positive Asymptomatic**	**36**		**36**		**100%**
**Negative**		**83**		**83**	**100%**
**Positive Percent Agreement**		**100% (98/98)**			
**Negative Percent Agreement**		**100% (83/83)**			

## Discussion

**Fig 2:**
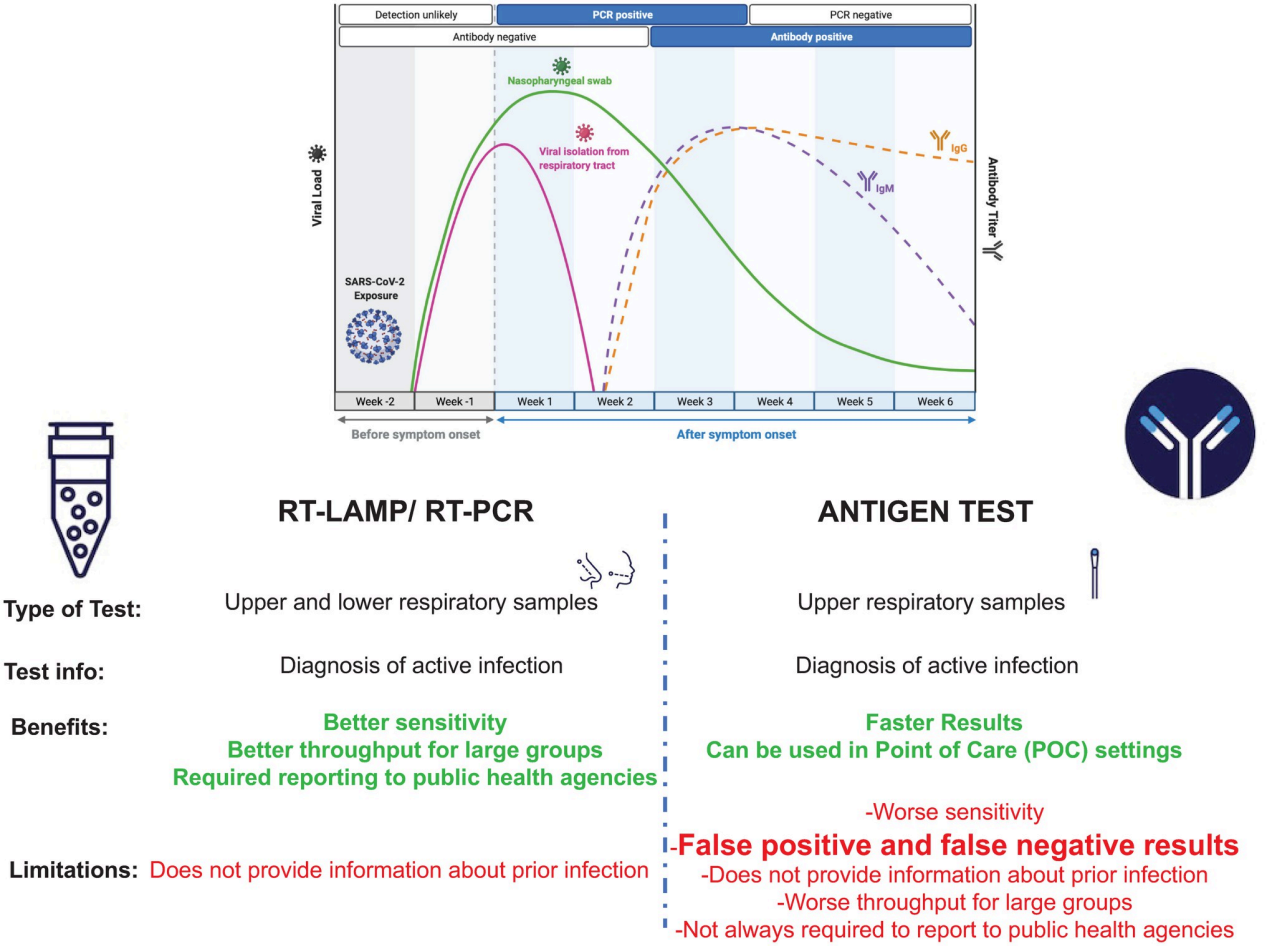
Comparison of RT-LAMP/PCR versus antigen tests for the detection of SARS-CoV-2.

According to the Center for Disease and Prevention (CDC) guidelines, upper respiratory specimens, including oropharyngeal and nasopharyngeal swabs, anterior nasal and mid-turbinate nasal swabs, nasopharyngeal washes/aspirates, or nasal aspirates as well as bronchoalveolar lavage (BAL), can be used for the detection of COVID-19 in healthcare settings. Commercial SARS CoV-2 diagnostic RT-PCR kits usually detect two or more genes related to the SARS-CoV-2 virus and require the classical experimental workflow where the sample is received in the laboratory, inventoried, RNA is extracted followed by RNA a quality control step, reverse transcription is performed, and PCR is performed. The entire process is not only labor-intensive (it can take up to more than 2 hours) but relies on expensive equipment (e.g., a quantitative PCR platform) and very often requires at least two optical filters to be able to read probes conjugated to two or more fluorophores. Additionally, the process relies on RNA extraction kits, plastic consumables, and trained laboratory scientists. Although ideal in a research laboratory setting, the entire pipeline has been revealed to be unrealistic in the context of the COVID-19 pandemic. Interestingly, a shortage of consumables and the lack of a trained workforce able to process laboratory specimens quickly and efficiently have afflicted laboratories worldwide, delaying testing. From a socioeconomic perspective, inequalities and disparities across countries have posed a challenge to COVID-19 testing. Many laboratories in challenging locations cannot afford expensive PCR equipment and highly trained staff. Rapid tests for COVID-19 based on antigen detection have been initially acclaimed as fast assays able to provide results in less than one hour, and in some cases, in less than 30 minutes. However, many concerns have been raised in the field due to collected data showing a continuous increase in false positivity rate and inaccuracies of these tests in some challenging circumstances [11,12]. Consequently, the FDA has issued an alert to healthcare providers regarding the potential for false-positive antigen results and steps to mitigate this risk (https://www.fda.gov/medical-devices/letters-health-care-providers/potential-false-positiveresults-antigen-tests-rapid-detection-sars-cov-2-letter-clinical-laboratory). Interestingly, concerns have also been raised related to false-negative results as a significant limitation of these tests. The local experience and reports to the FDA have found that antigen tests in symptomatic people are less sensitive than initially reported. In addition, these tests have much lower sensitivity when testing asymptomatic subjects. Rapid antigen tests can help quickly identify patients early in the course of SARS-CoV-2 infection when viral load is highest and who pose the greatest risk of SARS- CoV-2 transmission to others. They perform best when there is a high pre-test probability of infection (e.g., symptoms consistent with COVID-19, recent exposure to a known cause, and living/working in a setting where a high proportion of persons are infected). Thus, alternative testing methods are critically needed based on fast and reliable approaches. However, it is imperative to ensure that new candidate tests can guarantee low false positive and negative rates and ensure good specificity and sensitivity. Our study describes an RT-LAMP-based process that can quickly identify the SARS-CoV-2 RNA in clinical specimens in less than 1 hour. Because the method is based on an isothermal step, it does not require expensive PCR equipment. It could be quickly executed on a regular thermal cycler combined with a plate reader or water bath combined with a plate reader. The assay uses a fluorescent dye, and the end-point visualization can be achieved using any instrument with the following wavelength capacity: excitation 470 ±10 nm, emission 520 ±10 nm. Because the assay does not use probes but is primers based only, the manufacturing process is faster and extremely versatile as oligonucleotides can be obtained from several suppliers promptly. Perhaps, the most significant advantage of the assay described within the study is that no RNA extraction is needed. When samples are collected in the iSWAB^™^ (Mawi DNA Technologies) extraction-less buffer, the viral RNA is released into the collection tube and immediately available for assessment. Samples stored in the iSWAB^™^ do not require refrigeration and are stable at room temperature for up to twenty-one days. Both reverse transcription and LAMP reactions occur at 65 degrees Celsius, and thus, no preincubation and enzyme activation steps are required. Additionally, the assay uses a set of primers targeting three genes of the SARS-CoV-2 virus (Orf1, E1, N2) and an endogenous (18S RNA) gene. Combining three target genes (SARS-CoV-2) into the same reaction tube ensures maximum coverage and a broad detection compared to assessments based on one gene only (e.g., N1, N2). The RT-LAMP product can be monitored in a real-time fashion by the intercalating dye emission of fluorescence, or the emission signal can be detected by fluorescence readers or plate readers as an end-point assay. The specificity and sensitivity of the assay showed a high level of agreement with a standard RT-PCR FDA-approved comparator assay. Another essential advantage of the assay is that samples are analyzed in single-plex. Therefore, the probes’ signal interference, the relative expression levels of targets (including endogenous controls), and the dynamic range of their expression (issues often observed in RT-PCR approaches) do not represent a concern. The Prime CovidDetect^™^ Rapid Detection kit based on LAMP (like PCR-based approaches) offers advantages compared to antigen tests. Negative results from a rapid antigen test are often required to be confirmed by a molecular test, and antigen tests are more likely to miss an active SARS-CoV-2 infection than molecular tests Fig 2.

**Fig 3:**
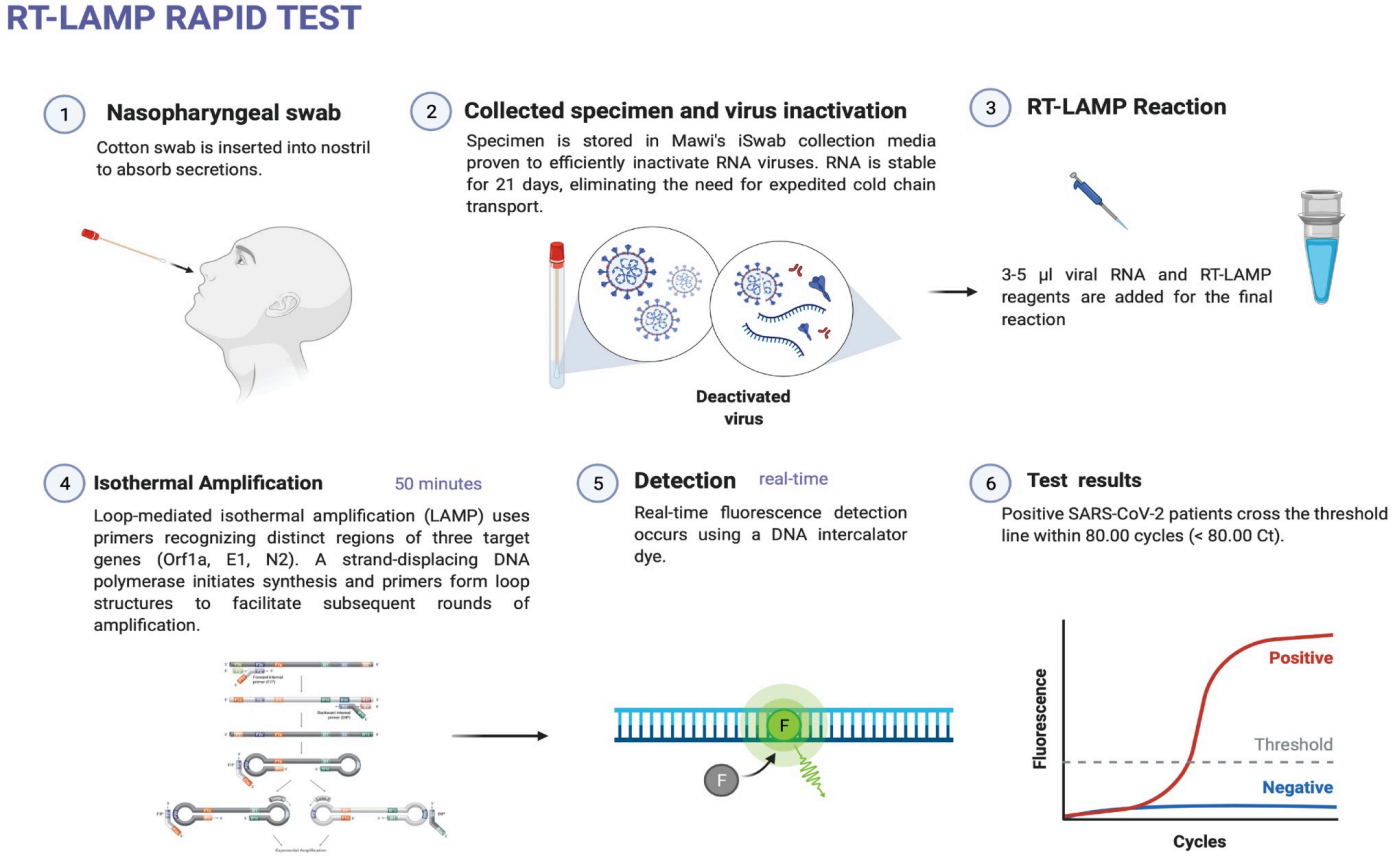
RT-LAMP rapid test workflow.

**Fig 4:**
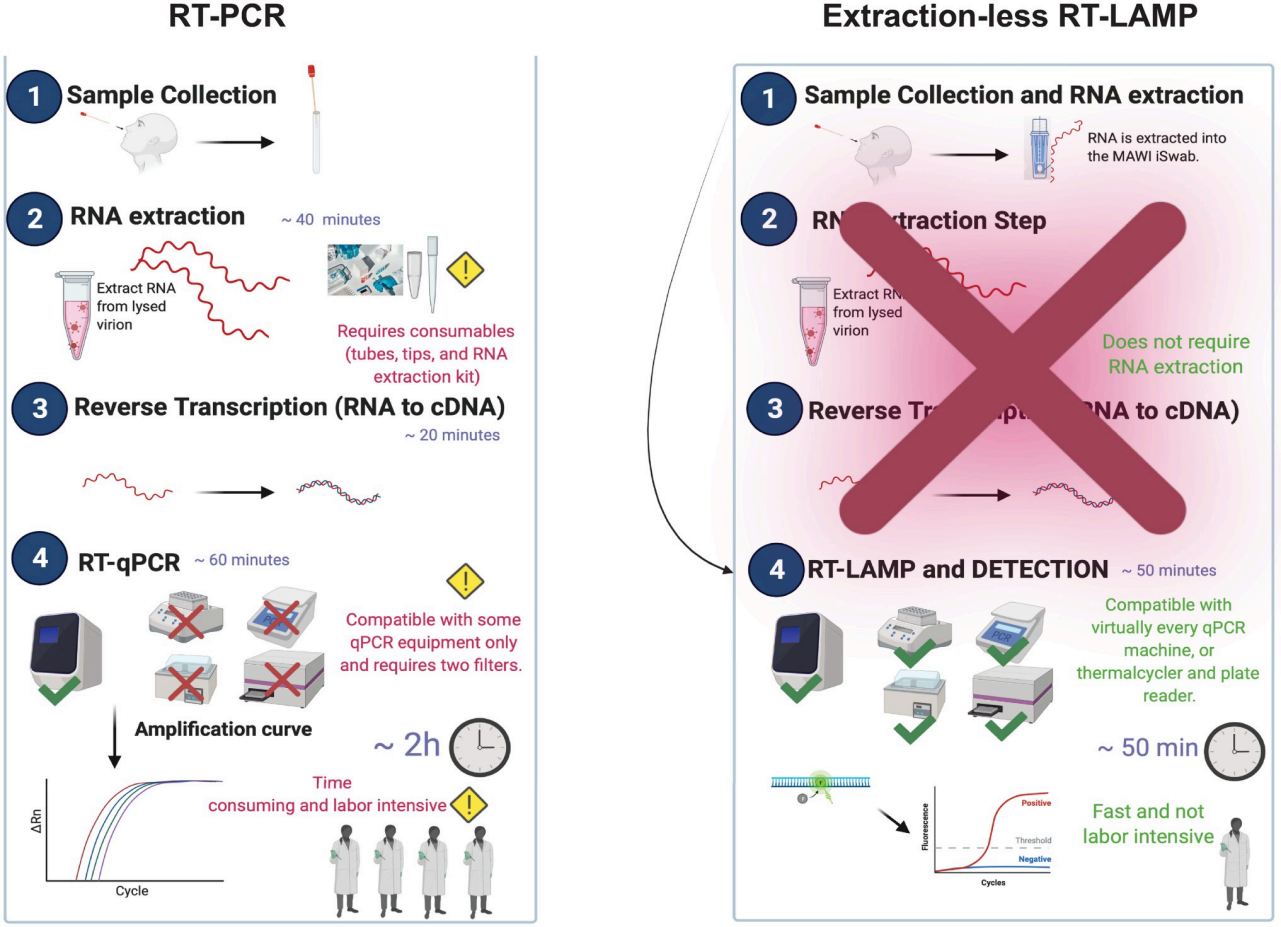
RT-PCR versus extraction-less RT-LAMP workflow.

Taken together, our data propose an entire pipeline (from sample collection to data visualization) that can efficiently be executed in less than 1 hour and presents a high level of versatility and adaptability not only to laboratory settings but also to impromptu testing sites Fig 3. Compared to a standard RT-PCR pipeline, the RT-LAMP assay provides a faster turnaround for data generation, is highly versatile, scalable on-demand, requires less workforce and presents advantages compared to rapid antigen tests Fig 4. Thus, making the assay a suitable candidate for SARS-CoV-2 detection in the context of the current COVID-19 pandemic. Additionally, data collected in our laboratory has shown that the same assay can be used for the detection of other pathogens like treponema pallidum, Influenza Viruses, Hepatitis virus, and more. Although the assay represents a valuable tool for the SARS-CoV-2 detection in clinical samples, significant limitations must be considered. The detection of 18S RNA indicates that human nucleic acid is present and implies that human biological material was collected, successfully extracted, and amplified. It does not necessarily suggest that the specimen is appropriate for detecting SARS-CoV-2.—Negative results do not preclude SARS-CoV-2 infection and should not be used as the sole basis for treatment. Optimum specimen types and timing for peak viral levels during infections caused by SARS-CoV-2 are not fully determined and might impact the assay. A false-negative result may occur if a specimen is improperly collected, transported, or handled.—If the virus mutates in the LAMP target regions, SARS-CoV-2 may not be detected.—Inhibitors and other types of interference may produce false-negative results—Detection of viral RNA may not translate to causation for clinical symptoms and severity of the symptoms.—The effect of vaccines, antiviral therapeutics, antibiotics, chemotherapeutic or immunosuppressant drugs has not been evaluated.

## Supporting information

S1 File Programming instructions, reaction set-up, and test interpretation. (DOCX)

S2 File Ct values of positive and negative samples. (XLSX)
