# Supplementary material for: A rapid, specific, extraction-less, and cost-effective RT-LAMP test for the detection of SARS-CoV-2 in clinical specimens
Source: PLoS One. 2022 Apr 11;17(4):e0266703. doi: 10.1371/journal.pone.0266703 (PMC9000105; doi:10.1371/journal.pone.0266703)
Supplement: S1 File — (DOCX) [file pone.0266703.s001.docx]

# **S1 File.**

**QuantStudio™ 3 and 5 Programming Instructions**

1. Turn on the instrument and wait for the instrument to initialize. Then switch on the computer/launch QuantStudio™ Design and Analysis Software v1.5.1.
2. Press the Create New Experiment button
3. Select the following settings

**Name**: Enter the plate name using the keyboard

**Barcode**: Assign the barcode number (if any)

**User name**: Enter the operator name

**Instrument Type**: Select the appropriate instrument [QuantStudio™](https://www.thermofisher.com/order/catalog/product/A34322) 3 System, or [QuantStudio™](https://www.thermofisher.com/order/catalog/product/A34322) 5 System

**Block Type**: Select the appropriate block type 96-well 0.1 ml Well Block, 96-well 0.2 ml Well Block, or 384-Well Block

**Chemistry**: Select Other

Run mode: select standard

1. Select the tab **Method**

**Volume**: Enter 10 μl, Cover 105.0 °C

**PCR Stage**: Select one PCR stage only, and assign the temperature to 65°C, time 00m:30s, and cycles 100x

**Data collection**: Select data collection ON

1. Select the tab **Plate** and select **Quick Setup**

Plate Attributes: Passive reference select ROX

1. Select the tab **Plate** and select **Advanced Setup**

**Targets**

Target 1: Name Orf1,E1, N2, Reporter FAM, Quencher NFQ-MGB

Target 2: Name 18s RNA, Reporter FAM, Quencher NFQ-MGB

**Samples**

Sample Name: Enter the sample name

Add comments and sample ID as needed.

1. Assign targets and samples to the plate visualized on the right of the screen
2. Select the Tab **Run** and click on **START RUN**
3. **Save the experiment** as needed and initiate the run
4. Upon completion of run, discard the plate in the biohazard waste according to the laboratory guidelines.

**QuantStudio™ 6 programming Instructions**

1. Turn on the instrument and wait for the instrument to initialize. Then switch on the computer/launch [QuantStudio™](https://www.thermofisher.com/order/catalog/product/A34322) Real Time PCR Software v1.7.1.
2. Press the Experiment setup button
3. Select the following settings on the Experiment Properties tab

**Experiment Name**: Enter the plate name using the keyboard

**Barcode**: Assign the barcode number (if any)

**User name**: Enter the operator name

**Instrument Type**: Select the appropriate instrument [QuantStudio™](https://www.thermofisher.com/order/catalog/product/A34322) 6 Flex System

**Block Type**: Select the appropriate block type 96-well 0.2 ml Well Block, or 384-Well Block

**Experiment**: Select Standard Curve

**Reagents**: Select Other

Run mode: select standard

1. Select the tab **Define**

**Targets**

Target 1: Name Orf1,E1, N2, Reporter FAM, Quencher NFQ-MGB

Target 2: Name 18s RNA, Reporter FAM, Quencher NFQ-MGB

**Samples**

Sample Name: Enter the sample name

Add comments and sample ID as needed.

**Passive Reference: Select ROX**

**Volume**: Enter 10 μl, Cover 105.0 °C

**PCR Stage**: Assign the temperature to 65°C, time 00m:30s, and cycles 100x

**Data collection**: Select data collection ON

1. Select the tab **Assign**

Assign targets and samples to the plate visualized on the right of the screen

1. Select the Tab **Run Method** and click on **START RUN**

**Volume**: Enter 10 μl, Cover 105.0 °C

**PCR Stage**: Select one PCR stage only, and assign the temperature to 65°C, time 00:30, and cycles 100x

**Data collection**: Select data collection ON

1. Select the tab **RUN**, save the experiment as needed and initiate the run
2. Upon completion of run, discard the plate in the biohazard waste according to the laboratory guidelines.

**Infinite Tecan 200 Pro™ programming Instructions**

The Veriti™ 96 or 384-Well Thermal Cycler is required when using the Infinite Pro™ plate reader only.

1. After preparing the master mix and adding the sample into each well, place the plate into the Veriti™ 96 or 384-Well Thermal Cycler and select an isothermal step.

**Volume**: Enter 10 μl, Cover 105.0 °C

**PCR Stage**: Assign the temperature to 65°C, time 40 minutes

1. Close the PCR machine lid and initiate the run. At the end of the run transfer the 96 or 384-Well into the Infinite Tecan 200 Pro™ plate reader.
2. Turn on the **Infinite Tecan 200 Pro™** and computer and wait until the instrument initializes.
3. Launch the **icontrol 2.0** software
4. Select the **Absorbance tab** and click on **default script**
5. Select the **plate definition tab** and select the appropriate plate type from the menu (e.g. for a 384-well plate select Corning 384 Flat Bottom Transparent Plate.
6. Select the number of wells used within the plate from the **Part of the Plate Tab.**
7. Select the **Fluorescence reading tab** and set the reading settings as follows

Mode: Fluorescence Top Reading

Excitation Wavelength: 485 nm

Emission Wavelength: 535 mm

Excitation Bandwidth: 20 nm

Emission Bandwidth: 25 nm

Gain: 10 manual (gain might be adjusted by the user according to the most appropriate settings. it is recommended to set a certain gain number and do not change is across plate prepared from the same stock of reagents e.g. dye)

Number of flashes: 25

Integration time: 20 μs

Lag time: 0 μs

Settle time: 0 ms

1. Move the instrument tray plate out
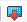
 and secure the plate into position
2. Move the instrument tray plate in
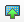
and start the reading
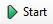

3. The instrument will generate automatically an excel file with the output of the reading.

**REACTION SET-UP**

| **SARS-CoV-2** | Volume Per Reaction in Microliters |  | **18S RNA** | Volume Per Reaction in Microliters |
| --- | --- | --- | --- | --- |
| Enzyme Mix | 4 |  | Enzyme Mix | 4 |
| Primers Mix (Assay) | 0.5 |  | Primers Mix (Assay) | -- |
| Primers Mix (Control) | -- |  | Primers Mix (Control) | 0.5 |
| Fluorescent Dye | 0.25 |  | Fluorescent Dye | 0.25 |
| DNA/RNA input + Water | 5.25 |  | DNA/RNA input + Water | 5.25 |

**TEST INTERPRETATION**

| **Sars-CoV-2**  (*ORF1*, E, N) | **18S RNA** | **Interpretation** | **Action** |
| --- | --- | --- | --- |
| Positive  2/2 Replicates | Positive  2/2 Replicates | SARS-CoV-2  **DETECTED** | Report results to physician, patient, and appropriate public health authorities. |
| Negative  2/2 Replicates | Positive  2/2 Replicates | SARS-CoV-2  **NON-DETECTED** | Report results to physician, patient, and appropriate public health authorities. |
| Negative  2/2 Replicates | Negative  2/2 Replicates | **FAILED** | Use residual clinical sample and repeat LAMP. If results remain FAILED, report to ordering physician and appropriate public health authorities. Report indicates that a new sample should be collected. |
| Positive  1/2 Replicates | Negative  1/2 Replicates | **INCONCLUSIVE** | Use residual clinical sample and repeat LAMP. If results remain INCONCLUSIVE, report to ordering physician and appropriate public health authorities. Report indicates that a new sample should be collected. |
